# Hip Arthrodesis with the Anterolateral Plate: An Innovating Technique for an Orphaned Procedure

**DOI:** 10.1371/journal.pone.0085868

**Published:** 2014-01-20

**Authors:** Patrick Hoekman, Garba Idé, Akambi Sanoussi Kassoumou, Mahamadou Malam Hayatou

**Affiliations:** Department of Orthopaedic Surgery, National Hospital of Niamey, Niamey, Niger, Africa; University of Michigan, United States of America

## Abstract

**Background:**

In developing countries hip osteoarthritis constitutes a major public health issue as it is highly prevalent in all age ranges of population, including the young. It often remains untreated because of the low accessibility of total hip prostheses. Hip arthrodesis still represents a major treatment option, but, for several reasons which are discussed in this paper, is nowadays infrequently performed. By means of reporting the results of a new simple technique, using a self-devised plate, the relevancy of hip arthrodesis in this particular environment is emphasized.

**Methods and Findings:**

Our series included 35 patients with painful hip osteoarthritis who underwent a hip fusion with the anterolateral arthrodesis plate. Two of them had a concurrent femoral osteotomy for correction of a vicious position of the limb and another patient had a femoral diaphysis osteotomy and placement of a Wagner elongating device in order to proceed with a limb lengthening by callotasis. The follow-up period averaged 16,9 months (9 to 34). All hips, except two, achieved solid fusion between 6 and 15 months after surgery. One failure of fusion was in the oldest patient, who presented a loosening of plate and screws due to an advanced degree of osteoporosis; the other was in a young patient who admitted having walked on his leg too soon. Patient satisfaction was high. We concluded that this technique is reliable and effective.

**Conclusions:**

The results of this study should convince the hesitant surgeon and patient to consider hip arthrodesis an acceptable treatment option for disabling hip arthritis, compared to no treatment at all.

## Introduction

Total hip prostheses are often too expensive and their availability too limited to cover the needs of patients with a painful osteoarthritic hip in developing countries. This is why in those regions of the world, hip arthrodesis remains a major treatment option. Various procedures have been described for hip fusion [1]–[10]. They are often complex, need a long period of post-operative immobilization [1], [5], [7]–[9], [11]–[13] and their failure and complication rate are not insignificant [1], [2], [5]–[7], [12], [14]–[20]. Improved results were obtained by techniques using plating techniques for stabilization, but these still remain intricate [10], [13], [18], [21]–[24].

To improve the image of hip arthrodesis (HA) and consequently to offer the opportunity to help patients who otherwise would remain untreated, the senior author (PH) has devised a new simple technique for HA based on a self-designed plate, the “anterolateral arthrodesis plate”.

We present the results of our experience with this method.

## Patients and Methods

### Ethic statement

The described procedure being only a variation of existing universally accepted principles and techniques for obtaining a hip arthrodesis by plating, ethical approval and written consent from the patients were deemed unnecessary. In conformity with the Declaration of Helsinki, art. 31, the authors have combined medical research with medical care, justified by the potential therapeutic value of the research and the certainty that participation in the research study would not adversely affect the health of the patients who serve as research subjects. Informed verbal consent by the patient was the prerequisite for considering the operation. The research has been integrally conducted in Niger, country of residence of all authors at the time of the study.

**Figure 1:**
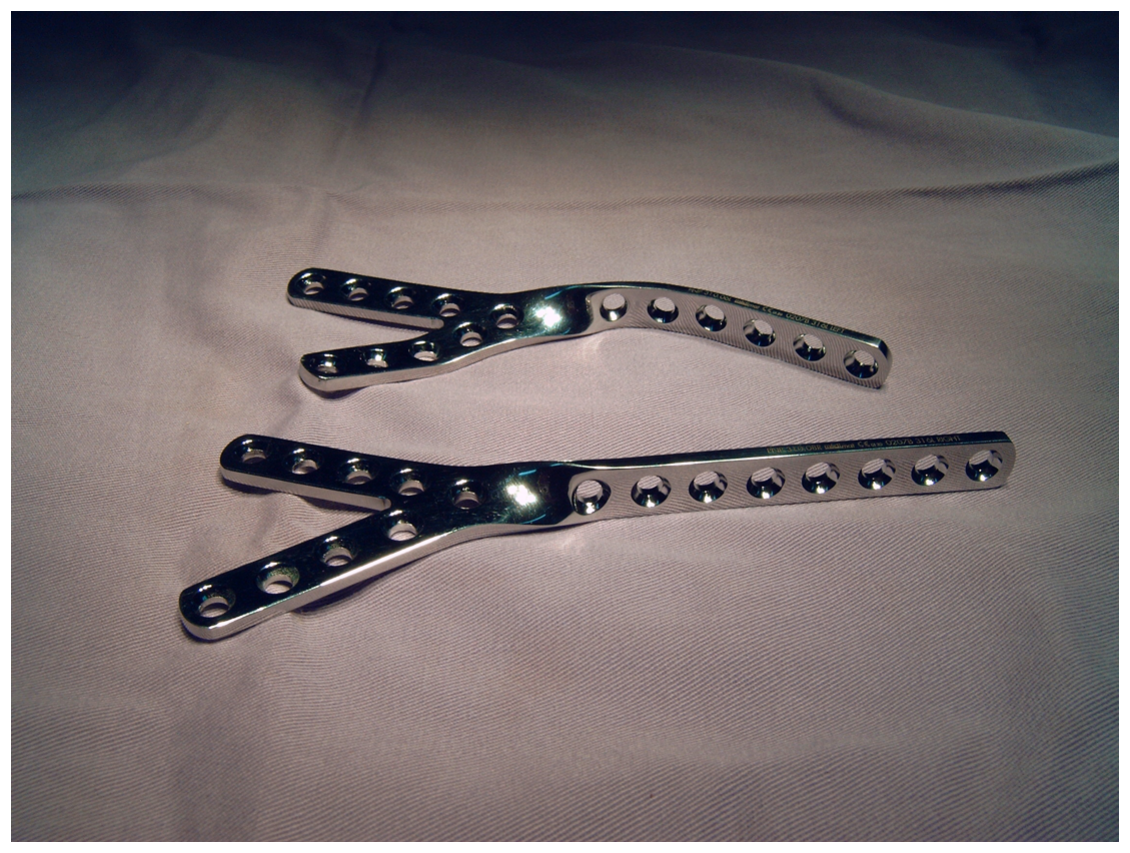
The anterolateral arthrodesis plate. Two different sizes of anterolateral arthrodesis plates are shown; one of the plates has been moulded into the appropriate shape to obtain the “arthrodesis position” of the leg.

Between 2006 and 2009, a HA was performed in Niger, a sub-Saharan African country, in 37 patients, using the anterolateral arthrodesis plate (Fig. 1). Two patients were lost to follow-up. As a result 35 patients form the basis of this study. The details of the patients are summarized in Table 1.

**Table 1:** Summary of the study group (n = 35 patients).

Criterion	Result	Number
Gender (%)	Male	23 (66)
	Female	12 (34)
Mean age in years (range)		26 (13 to 68)
Diagnosis (%)	Primary arthritis	3 (8.6)
	Slipped capital epiphysis (neglected)	3 (8.6)
	Trauma (neglected acetabular fractures)	7 (20.0)
	AVN* due to sickle-cell disease	9 (25.7)
	Tuberculosis of the hip	13** (37.1)
Mean follow-up time in months (range)		16,9 (9–34)

AVN: avascular necrosis of the femoral head.

Four with active TB.

Indication for surgery was a unilateral arthritis of the hip with intractable pain, refractory to non-operative treatment and restricting ambulation and activities of daily living. Considered not eligible for HA were patients with a diseased contralateral hip, an active pyogenous infection or a negative psychological attitude towards HA. After detailed information about what to expect of the procedure, a verbal consent from the patient was the prerequisite for the operation. The difficulties concerning religious kneeling and squatting were especially emphasized.

To compare pre- and post-operative status, clinical evaluation was based on those criteria of the Merle d'Aubigné hip score [25], which prove relevant in a HA context (pain and ability to walk) and on the measurement of any existing limb-length discrepancy (Table 2). Radiological evaluation was based on a standardized anterior-posterior radiograph of the hip and the pelvis and a lateral radiograph of the hip.

**Table 2:** Pre- and post-operative evaluation of the patients.

Evaluation criterion		Pre-operative	Post-operative
Merle d'Aubigné hip score criteria*	Pain	1.5	5.8
	Ability to walk	4.1	5.0
	Hip mobility	2.5	0
Limb-length discrepancy in cm (range)	All cases	3.0 (1 to 7)	
	Lengthened case excluded	2.7 (1 to 5)	3.6 (2 to 6)

Each criterion is rated from 0 points (worst condition) to 6 points (best condition).

If active tuberculosis of the hip was diagnosed on clinicoradiological basis, the patient was considered operable after a 2 months antituberculous treatment course with rifampicin, isoniazide and pyrazinamide. Treatment was continued for a total of 6 months, the last 4 months without pyrazinamide [26].

Patients were evaluated post-operatively every 3 months until radiographic fusion was evident. Radiographic fusion was defined by the disappearance of the hip joint space and its crossing by bone trabeculae. Failure of fusion after two years was considered a nonunion.

Any limb-length discrepancy was maintained at less than 2 cm by a shoe-lift to avoid low back pain and quadriceps deficiency later on [17]. Patient satisfaction and functionality were surveyed.

### Operative technique

**Figure 2:**
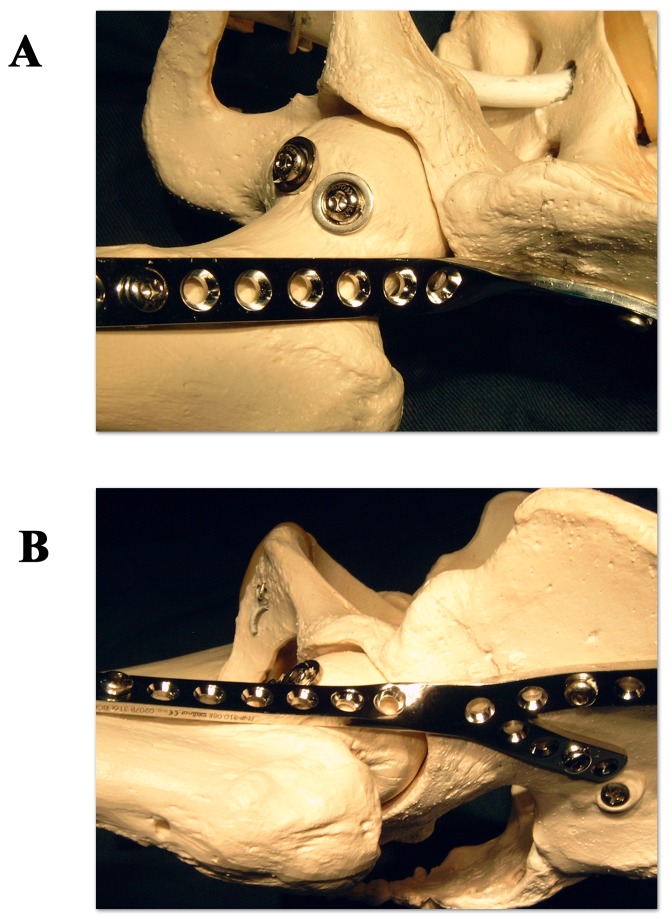
The different steps of the hip arthrodesis procedure with the anterolateral arthrodesis plate. The different steps of the procedure of the hip fusion with the anterolateral arthrodesis plate are depicted: a) transfixion of the femoral head and the acetabulum by two short sleeved 6.5 mm cancellous screws with washer and anterior view of the positioned anterolateral arthrodesis plate, b) lateral view the positioned anterolateral arthrodesis plate.

The patient is placed in a supine position with a small support under the affected hip. The iliac crest and the entire leg of the affected side are sterilely prepared and draped to allow complete mobility of the leg. A Smith-Petersen approach is used. The incision includes the anterior and middle third of the iliac crest, curves laterally distal to the anterosuperior iliac spine and proceeds parallel to the femur to 10 cm distally or more, if a concomitant corrective femoral subtrochanteric osteotomy is required to correct the hip and leg position. The fascia lata is incised and a plane between the tensor fascia muscle and the sartorius muscle is developed. If the lateral cutaneous femoral nerve is identified, it is retracted in medial direction. Care is taken not to go astray medially to the sartorius; this could endanger the femoral nerve and artery. To avoid this error, it is advised to check that the plane being developed has a muscle structure on each side, as no muscular structure exists medially to the sartorius. The tensor fascia lata muscle is detached subperiosteally from the iliac crest. The fascia layer between the tensor muscle and the rectus muscle is incised longitudinally as well as the fascia layer between the rectus and the vastus lateralis muscle. The lateral circumflex vessels are identified and transsected after ligation. The reflected head of the rectus femoris is separated from the joint capsule and the direct head detached from the anteroinferior iliac spine. The muscle is retracted medially. The vastus lateralis muscle is detached from its anterolateral bony origin and the proximal anterior portion of the femoral shaft exposed. An anterior and superior capsulectomy of the hip joint is performed, which provides a good visualisation of the femoral head and of the brim of the acetabulum, and allows the correction of any existing malposition of the hip. The femoral head is dislocated anteriorly, the ligamentum teres resected and the articular cartilage removed from head and acetabulum. An acetabular reamer can prove useful for the acetabular component. The joint must be completely debrided down to viable bone. If a congruency default is detected between femoral head and acetabulum due to a bone defect, it should be filled by cancellous bone. The graft can be taken from the iliac crest, taking care not to jeopardize the later screw fixation of the arthrodesis plate. The leg is positioned in the correct “arthrodesis position” for the hip: 20° of flexion, 5° to 10° of external rotation and 0° of abduction[17], [23], [27]. If this is not achievable, a corrective subtrochanteric osteotomy should be performed. Once the correct position is obtained and maintained, the femoral head is transfixed from its inferior pole into the acetabular bone in a cranial direction. Depending on the size of the femoral head, one or two short sleeved 6.5 mm cancellous screws with a washer are used for this purpose. The position of the screws should be as vertical as possible along the longitudinal axis of the body to obtain a maximal dynamic compression effect (Fig. 2 A). At this phase, the screw fixation is checked by moving the leg gently upwards and downwards; no movement should be perceived at articulation level. Movement still detectable translates an incorrect positioning of one or both screws. Once the fixation of the articulation is obtained and depending on the side which is operated on, a left or right anterolateral arthrodesis plate (Medimat-Exphar, Rixensart, Belgium) is chosen. It is bent into the required shape to accommodate the outline of the lateral face of the pelvic bone (V-side of the plate), the anterior side of the femoral neck, the proximal metaphysis and the proximal shaft of the femur (Figs. 1 and 2 A, B). The plate is available in different lengths and the chosen length will depend on the size of the bony structures and on the eventuality of an additional femoral corrective osteotomy. Fixation of the plate is performed by 4.5 mm cortical screws. The wound is closed on suction drainage.

Progressive weight bearing is allowed after 3 months if the radiographic signs of fusion are conclusive. The plate should be removed after 2 or 3 years to prevent a subjacent fracture of the femur [7].

The mean duration of the operation was 140 minutes (90–185) and the mean blood loss 350 cc (200–500).

## Results

In all patients the hip flexion could be corrected per-operatively to standards for hip arthrodesis position, except in two who required a concurrent femoral subtrochanteric osteotomy in order to correct the vicious position of the limb. In 7 patients iliac cancellous bone grafts were added in the hip joint space because of some incongruity between femoral head and acetabulum. In one patient with a 70 mm shortening of the leg, a femoral diaphysis osteotomy was performed with placement of a Wagner elongating device in order to proceed with a limb lengthening by callotasis.

One patient had a superficial incisional infection, which healed uneventfully under antibiotics. All patients left the hospital within 2 weeks after the operation.

**Figure 3:**
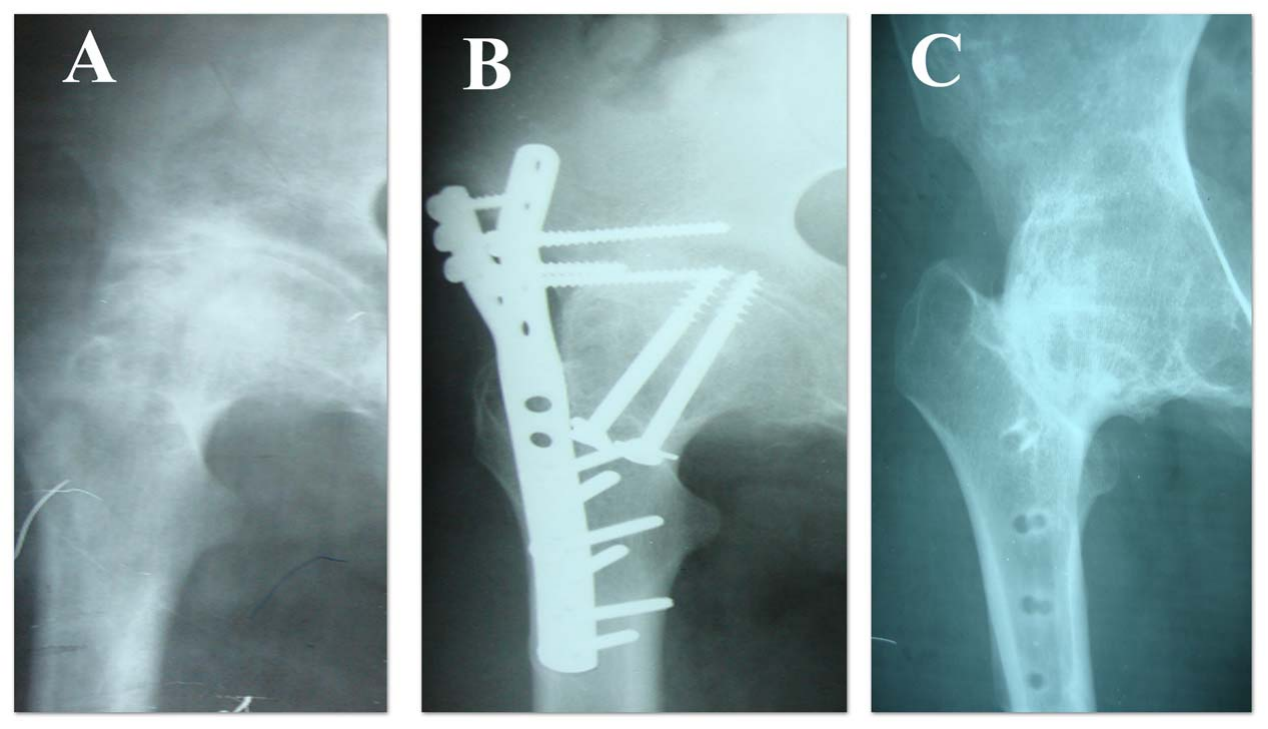
Radiological course of a hip fusion case with the view the anterolateral arthrodesis plate. Radiographs of the right hip in a 35 years old man showing a) a Kellgren 4 hip arthritis secondary to a neglected slipped capital epiphysis, b) the HA with the anterolateral plate 3 months post-operatively, c) fusion of the hip joint and removal of the plate 25 months post-operatively.

All 35 hips, we were able to follow, fused to an arthrodesis, except two which evolved to a nonunion, one of these had a concurrent material breakage. As both nonunions were painless, they were left undisturbed. The mean fusion rate was 10,5 months (6–15 months). No further shortening of the leg exceeding 10 mm was recorded (Table 2). The distraction by callotasis resulted in a limb lengthening of 50 mm and the Wagner device was removed after 8 months. Three plates were removed uneventfully 2 years post-operatively during the period of the study (Fig. 3).

Based on the Merle d'Aubigné hip score criteria, the average pain score improved from 1.5 to 5.8, the ambulation from 4.1 to 5.0, and the limb-length discrepancy had a mean increase of 9 mm (Table 2). Only one patient was dissatisfied. All the other patients rated their quality of life as “good” to “very good” and would undergo the same operation again. Two patients got pregnant during follow-up and the delivery was uneventful.

## Discussion

Thirty of our 35 patients (85,7%) were under 40 years of age. Because of its specific etiological spectrum (see Table 1), the population affected by osteoarthritis of the hip in sub-Saharan Africa is usually young and active [29]. This leads to a high prevalence of hip arthritis in this portion of the population, with an ensuing significant burden of disease [30] and a resulting deleterious effect on economics.

To deal with the osteoarthritic hip joint, besides femoral and pelvic osteotomies, which have proven useful on the short term but generally are not a final solution to the problem, two main treatment options subsist: total hip arthroplasty (THA) and hip arthrodesis.

In Niger, the availability of THA turns out to be very limited. In this socioeconomically constrained country, hip prostheses can only be purchased at high prices or are very occasionally offered free of charge by the government or by some non-governmental organization. Furthermore, in this context, the efficiency of THA discloses to be quite questionable: not only THA is known to be coupled with a higher failure rate in young active patients [16], [31]–[34], but an ethical issue turns up as well, as a subsequent revision arthroplasty after complication or after wear cannot invariably be guaranteed. In some developing countries audits on THA performance have recently started [29], but no long term follow-up studies were found for sub-Saharan Africa.

When patients cannot afford a THA or when it is not available, the remaining operative option remains HA. The problem regarding HA, however, is that in this joint prosthesis era, it is currently only seldom performed in adolescents and adults [24], [35]. In developing countries, however, where hip tuberculosis is prevalent, it is still occasionally performed in children [36].

Despite the high prevalence of patients who consult at our hospital for hip arthritis, only 37 patients underwent a HA in a four years period. One explanation for this relative scarcity is the comprehensive reluctance of the patient for a procedure which abolishes hip mobility. That hesitancy is enhanced by the persisting theoretical hope of ever obtaining a hip prosthesis, in spite of the awareness of its low likelihood and of the long-term drawbacks linked to the young age of the patients. Another reason is probably some diffidence from many surgeons regarding HA, mostly due to an insufficient experience in the functional results of HA and a lack of training in performing HA. «The most acid critics of arthrodesis of the hip joint are those who never do it» (Sir Reginald Watson-Jones) [6].

Both above mentioned reasons, which lead to a situation where many patients with a painful hip arthritis remain untreated, have incited us to promote HA by proposing a simple technique and restore its lost credibility by publishing its results.

The procedure for HA, described in this study, is based on the fixation and intrinsic compression of the femoroacetabular components by means of two lagscrews, subsequently reinforced by a neutralization plate, the “anterolateral plate”. It resulted in a 94% fusion rate. Two nonunions occurred, one caused by loosening of the plate and the other due to material breakage. The first was in the oldest patient (68) who presented an advanced osteoporosis and the second in a young patient (20) who admitted having walked on his leg without crutches immediately after leaving the hospital The main reason for patient satisfaction was the disappearance of hip pain after the operation and the resulting greater ability to walk (Table 2). Patients stated that these benefits outweighed the disadvantages of hip movement abolition and its ensuing problems with kneeling for praying and with squatting for bowel movement. The one unsatisfied patient was a woman of 24 with a sound fusion after operation, who had misunderstood a crucial issue of the procedure, namely the abolition of hip movement. She was reassured by the prospect of the existing possibility of desarthrodesis at a later stage, if her discontentment persisted.

Compared to other plating techniques, the fusion rate of this technique is higher than the procedure making use of the “ventral plate” (83%) [5] and comparable to the HA technique using the “cobra plate” (90 – 100%) [9], [28]. However, the anterolateral plate technique proves easier to perform and a major advantage is the preservation of the hip abductor muscles and of the greater trochanter, allowing desarthrodesis later on [33], [37]–[42]. Other advantages are the maximal preservation of bone stock, the applicability of the procedure in older children, the possibility of performing other procedures, like osteotomies, simultaneously and the fact that no obligatory roentgenographic control is required during the procedure, as the whole operation can be performed under direct vision.

Additional to a diseased contralateral hip and a pyogenous infection, osteoporosis should be considered a contraindication for the procedure as well.

In conclusion, the purpose of this study was not to make an apology for HA against contemporary THA, which has indisputably proven its efficiency [43], [44], even in hip tuberculosis [45]. The occurrence in slightly more than fifty percent of the patients of low back pain and ipsilateral knee pain many years after operation are well known long terms effects of HA [16], [17], [19], [23], [24], [27], [33], [46]–[48]. The main goal was to demonstrate that with this simple, cheap and reliable technique HA is still a very valuable option for painful hip arthritis in countries where THA accessibility is low. The high fusion and patient satisfaction rate, ensuing from this technique for HA, and the prospect of reversibility of the HA into a THA by means of a desarthrodesis, should convince the indecisive surgeons and patients to consider HA an acceptable treatment option, compared to no treatment at all.
